# *tert*-Butylhydroquinone Treatment Alleviates Contrast-Induced Nephropathy in Rats by Activating the Nrf2/Sirt3/SOD2 Signaling Pathway

**DOI:** 10.1155/2019/4657651

**Published:** 2019-12-18

**Authors:** Qin Zhou, Xin Wang, Xiaofei Shao, Honglei Wang, Xiaobo Liu, Xiaosu Ke, Chongxiang Xiong, Lixin Wei, Hequn Zou

**Affiliations:** ^1^Department of Nephrology, The Third Affiliated Hospital of Southern Medical University, Guangzhou, Guangdong 510630, China; ^2^Department of Nephrology, Fujian Medical University Union Hospital, Fuzhou, Fujian 350001, China

## Abstract

Oxidative stress plays a critical role in the pathophysiology of contrast-induced nephropathy (CIN). Since the specific treatment of CIN remains an unmet medical need, it is imperative to find an effective strategy against the clinical management of CIN. The transcription factor Nrf2 is known to regulate antioxidative stress response. The aim of the present study was to assess the effects of *tert*-butylhydroquinone (t-BHQ), an activator of Nrf2, in the prevention of CIN and elucidate the underlying mechanism of its action *in vitro* and *in vivo*. We established a rat model of CIN and treated the animals with t-BHQ (25 mg/kg). The effects of t-BHQ treatment on CIN rats were elucidated by assessing renal function, HE staining, immunohistochemistry, and western blotting. We also studied the activity of oxidative stress-related markers, such as intracellular ROS level, MDA level, SOD2 activity, and GSH/GSSG ratio. We validated our results by siRNA-mediated silencing of Nrf2 in HK-2 cells exposed to the radiocontrast agent. Treatment with t-BHQ significantly ameliorated the renal function and the histopathological lesions in CIN rats. Further, pretreatment with t-BHQ significantly increased the SOD2 activity and GSH/GSSG ratio and decreased the levels of ROS and MDA in animals subjected to ioversol exposure. In addition, t-BHQ treatment increased the expression of Nrf2, Sirt3, and SOD2 and concomitantly decreased the expression of acetylated-SOD2. When Nrf2-silenced HK-2 cells were exposed to radiocontrast agent, they suffered severe cell oxidative stress, exhibited lower expression of Sirt3 and SOD2, and expressed higher levels of acetylated-SOD2; however, t-BHQ treatment did not affect the protein expression of these indicators in si-Nrf2 HK-2 cells. Our findings suggested that Nrf2 plays an important role in the regulation of the Sirt3/SOD2 antioxidative pathway, and t-BHQ may be a potential agent to ameliorate radiocontrast-induced nephropathy via activating the Nrf2/Sirt3/SOD2 signaling pathway in vitro and in vivo.

## 1. Introduction

Radiocontrast agents are widely used in invasive image examinations such as contrast-enhanced CT and angiography. However, millions of patients who undergo radio imaging procedures are at the risk of contrast-induced nephropathy (CIN). CIN has become the third leading cause for hospital-acquired acute kidney injury and associated mortality [1]. In a meta-analysis of 29 studies where patients were injected with the radiocontrast medium either intravenously or intra-arterially, the incidence of CIN was observed to be between 4.4% and 22.1% [2]. CIN is defined as a rise in serum creatinine level ≥ 0.5 mg/dL or a ≥25% relative rise in creatinine level from baseline within 48 hours of exposure to the contrast agent, accompanied by an otherwise unexplained acute impairment in renal function [3].

The pathogenesis of CIN is not completely known, and therefore, there is no specific treatment for the clinical management of CIN. However, renal parenchymal hypoxia and the generation of reactive oxygen species (ROS) have been reported to play a critical role in the pathogenesis of CIN [4]. Studies based on animal models of CIN have shown that the disturbance of oxygen balance may result in cell apoptosis and necrosis and may manifest as medullary hypoxic injury [5].

Sirtuins belong to the family of conserved nicotinamide adenine dinucleotide- (NAD^+^-) dependent deacetylases and are involved in stress response, cell proliferation, and antiapoptosis processes [6]. Sirtuin-3 (Sirt3) is localized mainly in the mitochondrial matrix [6]. It plays a pivotal role in maintaining mitochondrial function, protecting against oxidative stress-induced damage by deacetylating lysine residues of mitochondrial proteins, and maintaining the kidney function [7]. Sirt3 eliminates ROS by transforming acetylated-SOD2 (Ac-SOD2) into SOD2 [8]. It regulates microtubule network-dependent trafficking of functional mitochondria between renal tubular epithelial cells, thereby preserving the cellular bioenergetic profile and sustaining the protection against oxidative stress [9]. We have previously reported that decreased expression of Sirt3 protein correlated with the early-stage chronic renal allograft dysfunction in a rat model [10]. However, little is known about the regulation of Sirt3 expression. The nuclear factor erythroid 2-related factor (Nrf2) is one of the crucial antioxidant proteins in the defense system against oxidative stress. As a transcription factor, it can regulate the expression of genes with a role in the antioxidant and cytoprotective role [11]. The NRF-2A subunit can directly bind to the Sirt3 promoter, and hence, the knockdown or overexpression of Nrf2 can modulate Sirt3 levels [12].

*tert*-Butylhydroquinone (t-BHQ), an agonist of Nrf2, is an aromatic organic compound with highly effective antioxidant function [13]. Since the specific treatment of CIN remains an unmet medical need, it is imperative to find an effective strategy against the clinical management of CIN. The aim of the present study was to investigate the protective role of t-BHQ against oxidative stress in CIN and to elucidate the underlying molecular mechanisms of its action.

## 2. Materials and Methods

### 2.1. Animals and Groups

Adult Sprague-Dawley rats weighing about 180-200 g were purchased from the animal center of Southern Medical University (Guangzhou, China). The animals were housed in a specific pathogen-free laboratory at the Animal Experimental Center of the Southern Medical University (Guangzhou, China) under standard conditions with controlled temperature (24 ± 2°*C*), humidity (40-60%), and a 12 h light/dark cycle. The animals were fed a standard rat chow diet and had free access to water. The study was approved by the Committee on the Ethics of Animal Experiments of the Southern Medical University and was carried out following the *Guide for the Care and Use of Laboratory Animals* published by the United States National Institutes of Health [14]. The rats were divided into (1) control group (*n* = 6), (2) ioversol group (*n* = 6), (3) ioversol+t-BHQ group (*n* = 6), and (4) t-BHQ group (*n* = 6). The CIN was established in the rat model as reported earlier [15]. Briefly, the rats were anesthetized with pentobarbital sodium, and the following drugs were sequentially injected in the tail vein: indomethacin (Sigma, USA) (10 mg/kg), followed by Nw-nitro-L-arginine methyl ester (L-NAME) (Hengrui Medicine, Ltd., Jiangsu, China) (100 mg/kg) and ioversol (Hengrui Medicine, Ltd., Jiangsu, China) (2.9 g/kg) 15 and 30 min later. The animals in the control group received subcutaneous injections of saline at each time point. The animals in the ioversol+t-BHQ group were gavaged with t-BHQ at the dose of 25 mg/kg for 5 days consecutively before CIN was established [16]. Rats in the t-BHQ group were gavaged with t-BHQ at the dose of 25 mg/kg for 5 days consecutively followed by 0.9% saline injection through the tail vein. The animals were sacrificed 24 h after 5 days with an overdose of pentobarbital sodium.

### 2.2. Tissue Harvesting

The kidneys were harvested from the animals of all the groups. Each kidney was cut into two sections: one section was stored in liquid nitrogen for western blot analysis and the second section was fixed in 4% formalin for the pathological examination and immunohistological staining.

### 2.3. Renal Function Analysis

Rat serum samples (isolated from 2 ml blood collected by heart perfusion) were used for the measurement of blood urea nitrogen and creatinine by an automatic biochemical analyzer (AU5400; Olympus, Tokyo, Japan).

### 2.4. Renal Histopathology and Immunohistological Staining

The renal tissues were fixed in 4% paraformaldehyde for 24 h and embedded in paraffin. Sections of 2 *μ*m thickness were cut, stained with hematoxylin and eosin (HE), and observed under a light microscope (Nikon Eclipse TE2000-U, NIKON, Japan) at 200x magnification to evaluate the extent of pathological changes in the kidney. For immunohistochemical staining, antigens were retrieved by the microwave antigen retrieval method using citric acid buffer (pH 6.0) and detected via PV-9001 staining (Golden Bridge Biotechnology Co., Beijing, China). Sections of 4 *μ*m thickness were cut, mounted on the glass slides, and incubated with rabbit polyclonal anti-Sirt3 (1 : 100, Cell Signaling Technology, USA) and rabbit polyclonal anti-Nrf2 (1 : 100, Abcam, Cambridge, MA) antibodies overnight at 4°C, followed by incubation with the goat anti-rabbit secondary antibody (1 : 100; Golden Bridge Biotechnology Co., China) for 20 min. The bands were visualized and positive staining was quantified using Image-Pro Plus software version 7.0.1.658, Media Cybernetics, Inc. (Rockville, MD, USA).

### 2.5. Assessment of Oxidative Stress

We used Cu/Zn-SOD and Mn-SOD Assay Kit with WST-8 (Beyotime Institute of Biotechnology, Shanghai, China) to measure SOD2 enzymatic activity according to the manufacturer's instructions by the method of Zeng et al. [17]. The intracellular ROS were estimated by detecting 2,7-dichlorofluorescein diacetate (DCFH-DA) (Sigma, USA). The levels of malondialdehyde (MDA) were evaluated by commercially available total superoxide dismutase and malondialdehyde assay kits (Nanjing Jiancheng Bioengineering Institute, China) according to the manufacturer's protocol. The intracellular levels of GSH were measured using GSH and GSSG Assay Kit (Beyotime Institute of Biotechnology, Shanghai, China) according to Sun et al. [18].

### 2.6. Cell Culture

Kidney tubular epithelial cells of human (HK-2) were purchased from ATCC, USA, and cultured in DMEM-F12 mixture (Gibco, USA) with 10% fetal bovine serum (Gibco, USA), 2 mmol/l L-glutamine, and 100 U/ml penicillin-streptomycin at 37°C and 5% CO_2_. HK-2 cells were treated with 50 mg/ml ioversol for 24 h to induce oxidative stress. Subsequently, the amount of intracellular reactive oxygen species (ROS) was assessed by dichloro-dihydro-fluorescein diacetate (DCFH-DA) staining using a commercially available kit (Nanjing Jiancheng Bioengineering Institute, China). Briefly, HK-2 cells were preincubated with 20 *μ*mol/l t-BHQ for 30 min before ioversol treatment [19]. Cell death was measured using the tetrazolium salt Cell Counting Kit-8 (CCK-8; Beyotime Institute of Biotechnology, China). We followed the methods of Li et al. [20]. For Nrf2 silencing, Lipofectamine 2000 (Invitrogen, Carlsbad, USA) was used to transfect the HK-2 cells with 50 nM of small interfering RNA (siRNA) for 24 h according to the manufacturer's instructions. The sequence of siRNA used to silence Nrf2 was sense 5′-GAG AAT TCC TCC CAA TTC AGC-3′; antisense 5′-TTT GGG AAT GTG GGC AAC-3′ (Shanghai GenePharma Co.). The efficiency of silencing was tested by western blot analysis. Finally, the Nrf2 knockdown cells were treated with 50 mg/ml ioversol for 24 h.

### 2.7. Western Blotting

For the immunoblot analysis, the total protein was extracted from the frozen kidney tissue and treated HK-2 cells using RIPA lysis buffer supplemented with protease and phosphatase inhibitors (Roche, USA). The protein concentrations of the samples were measured using the BCA Protein Assay Kit (Beyotime Institute of Biotechnology, Shanghai, China). The nuclear and cytoplasmic proteins fractions were extracted using the respective kits according to the manufacturer's instructions (Beyotime Institute of Biotechnology, Shanghai, China). Protein samples were separated by electrophoresis and transferred to PVDF membranes (EMD Millipore, Billerica, MA, USA). Subsequently, the membranes were washed thrice with Tris-buffered saline-Tween (TBST) and blocked with 5% bovine serum albumin (Sigma-Aldrich, Merck KGaA) in TBST for 1 h at 4°C. Next, the membranes were incubated with the primary antibodies against Nrf2 (dilution 1 : 1000, Abcam, Cambridge), SIRT3 (dilution 1 : 1000, Cell Signaling Technology, USA), SOD2 (dilution 1 : 500, Abcam, Cambridge), and Acetyl-Mn-SOD K68 (dilution 1 : 1000, Abcam, Cambridge) overnight at 4°C. The membranes were washed and incubated with the secondary antibody (dilution 1 : 5000, CW Biotech, China) for 1 h at room temperature. The target bands were observed by the SuperSignal West Pico enhanced chemiluminescent substrate (Pierce; Thermo Fisher Scientific, Inc., Waltham, MA, USA), and quantified by ImageJ software (version 1.46, National Institutes of Health, Bethesda, MD, USA). All experiments were repeated in triplicates.

### 2.8. Statistical Analysis

The data are presented as the means ± standard error (SE). Student's *t*-test or one-way analysis of variance (ANOVA) was performed to analyze the significance of differences in multiple group comparisons using the SPSS software 13.0. The statistical significance was set at *P* < 0.05 and *P* < 0.01.

## 3. Results

### 3.1. t-BHQ Ameliorates CIN-Associated Renal Dysfunction and Renal Histological Damage in CIN Rats

**Figure 1:**
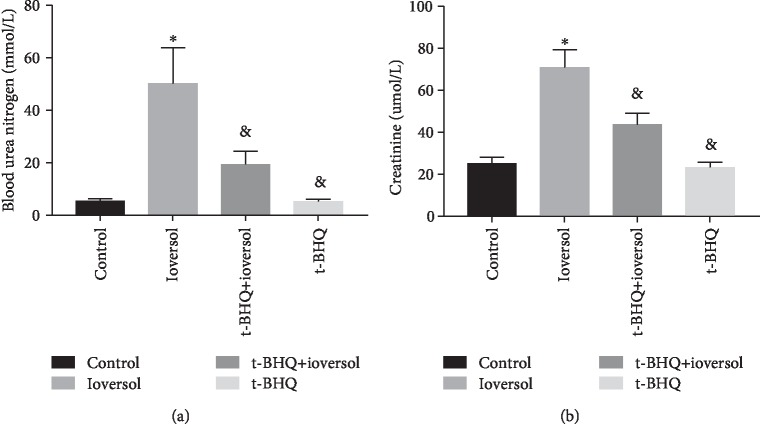
t-BHQ decreased blood urea nitrogen (a) and serum levels (b) in CIN rats. Rats in the ioversol group were sequentially injected in the tail vein: indomethacin (10 mg/kg), followed by L-NAME (100 mg/kg) and ioversol (2.9 g/kg) 15 and 30 min later. t-BHQ was administered to rats in the t-BHQ+ioversol group. Date was expressed as the mean ± SD. ^∗^*P* < 0.01 versus the control group; ^&^*P* < 0.05 versus the ioversol group.

**Figure 2:**
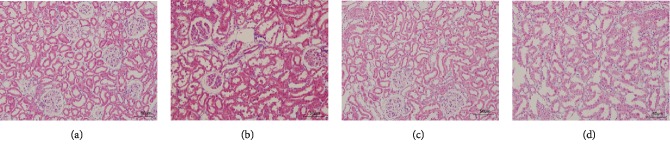
t-BHQ alleviated histological change: (a) control group; (b) ioversol group; (c) t-BHQ+ioversol group; (d) t-BHQ group. Rats in the ioversol group were sequentially injected in the tail vein: indomethacin (10 mg/kg), followed by L-NAME (100 mg/kg) and ioversol (2.9 g/kg) 15 and 30 min later. t-BHQ was administered to rats in the t-BHQ+ioversol group.

As shown in Figure 1, levels of serum and blood urea nitrogen were significantly increased in animals treated with ioversol compared with the control group (*P* < 0.01). However, the levels reduced significantly following treatment with t-BHQ (*P* < 0.05). These results were supported by the pathological alterations seen in animals. As shown in Figure 2, animals treated with ioversol showed severe pathological alterations in the kidneys, including luminal congestion, renal tubular necrosis, and vacuolar degeneration. However, the lesions improved significantly following treatment with t-BHQ. These results indicated that t-BHQ may have a renoprotective role.

### 3.2. t-BHQ Alleviated CIN-Induced Oxidative Injury in CIN Rats

**Figure 3:**
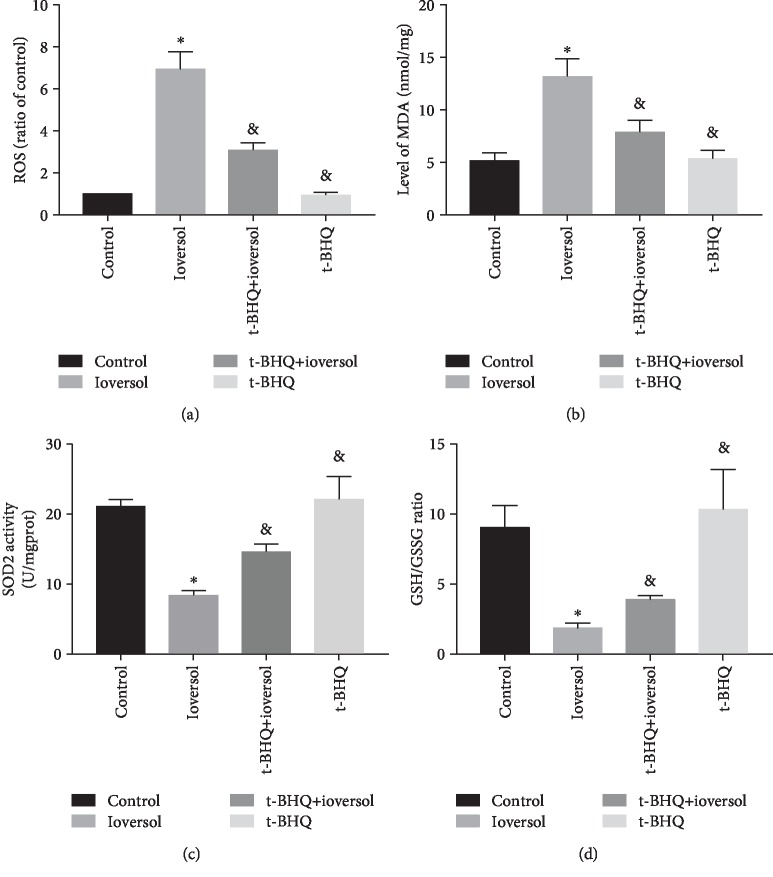
Effects of t-BHQ on CIN-induced oxidative stress in the kidney of CIN rats. (a) ROS level. (b) Malondialdehyde (MDA) level. (c) SOD2 activity. (d) GSH/GSSG ratio. Rats in the ioversol group were sequentially injected in the tail vein: indomethacin (10 mg/kg), followed by L-NAME (100 mg/kg) and ioversol (2.9 g/kg) 15 and 30 min later. t-BHQ was administered to rats in the t-BHQ+ioversol group. Data were expressed as the mean ± SD. ^∗^*P* < 0.01 versus the control group; ^&^*P* < 0.05 versus the ioversol group.

To explore the effect of t-BHQ on CIN-induced oxidative injury, we measured the levels of ROS, MDA, SOD2 activity, and GSH/GSSG ratio in treated animals. Our results showed that contrast agent exposure caused increase in intracellular ROS (Figure 3(a)); animals treated with ioversol showed lower SOD2 activity (Figure 3(c)) and GSH/GSSG ratio (Figure 3(d)) and a higher MDA content (Figure 3(b)) than the control group (*P* < 0.01). However, these observations were reversed in animals pretreated with t-BHQ (*P* < 0.05).

### 3.3. t-BHQ Treatment Enhanced Nrf2 Nuclear Translocation and Increased Sirt3 Expression in CIN Rats

**Figure 4:**
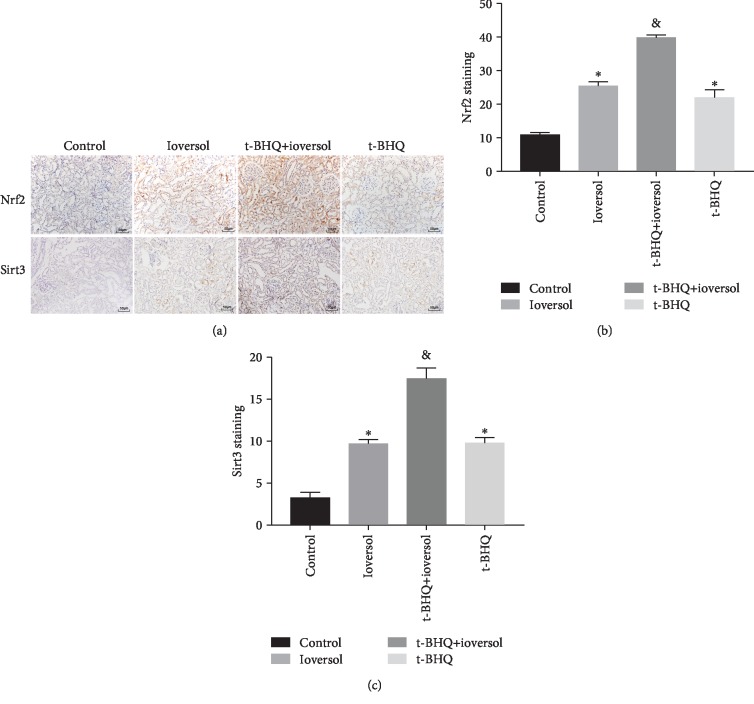
Immunohistochemical staining and semiquantitative analysis of Nrf2 and Sirt3 in the kidneys of different groups. (a) Immunohistochemical staining of Nrf2 and Sirt3. (b) Quantitative analysis of Nrf2. (c) Quantitative analysis of Sirt3. Original magnification ×200. Rats in the ioversol group were sequentially injected in the tail vein: indomethacin (10 mg/kg), followed by L-NAME (100 mg/kg) and ioversol (2.9 g/kg) 15 and 30 min later. t-BHQ was administered to rats in the t-BHQ+ioversol group. Data were expressed as the mean ± SD. ^∗^*P* < 0.01 versus the control group; ^&^*P* < 0.05 versus the ioversol group.

**Figure 5:**
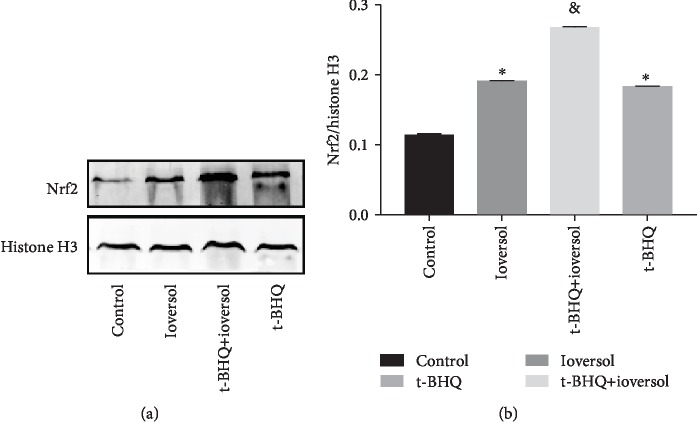
Western blot (a) and quantitative analysis of Nrf2 in rats (b). Rats in the ioversol group were sequentially injected in the tail vein: indomethacin (10 mg/kg), followed by L-NAME (100 mg/kg) and ioversol (2.9 g/kg) 15 and 30 min later. t-BHQ was administered to rats in the t-BHQ+ioversol group. Data were expressed as the mean ± SD. ^∗^*P* < 0.05 versus the control group; ^&^*P* < 0.05 versus the ioversol group.

**Figure 6:**
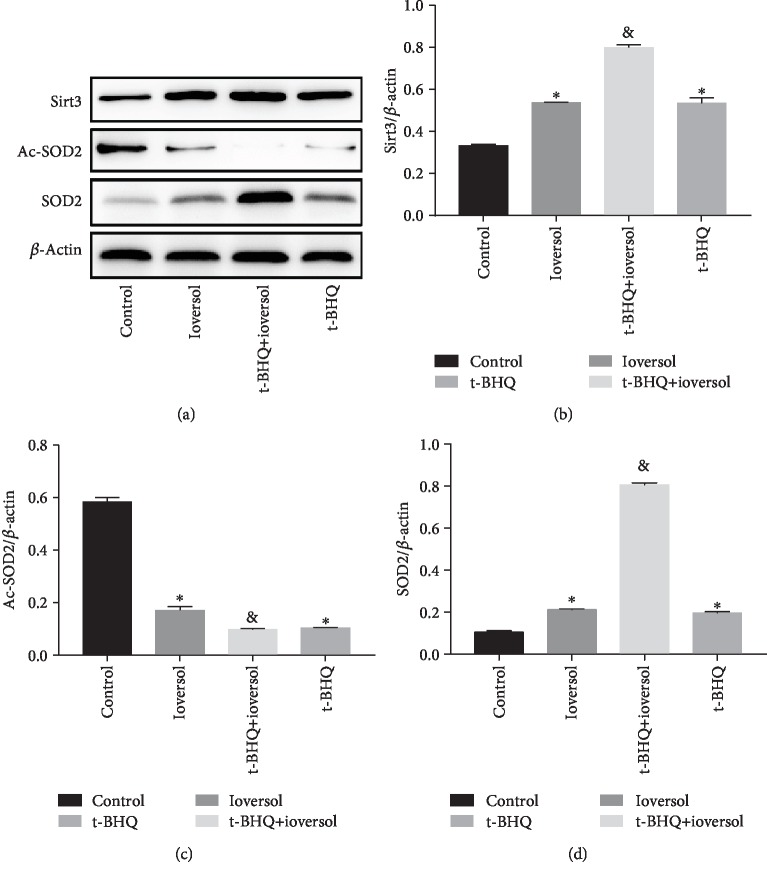
The protein levels in different groups. (a) Western blot of Sirt3, Ac-SOD2, and SOD2. (b) Quantitative analysis of Sirt3. (c) Quantitative analysis of Ac-SOD2. (d) Quantitative analysis of SOD2. Rats in the ioversol group were sequentially injected in the tail vein: indomethacin (10 mg/kg), followed by L-NAME (100 mg/kg) and ioversol (2.9 g/kg) 15 and 30 min later. t-BHQ was administered to rats in the t-BHQ+ioversol group. Data were expressed as the mean ± SD. ^∗^*P* < 0.05 versus the control group; ^&^*P* < 0.05 versus the ioversol group.

To elucidate the protective mechanism of t-BHQ in CIN-induced oxidative stress in the rat model, we measured the protein expression level of Nrf2, Sirt3, Ac-SOD2, and SOD2. Our results from immunohistochemistry showed that the levels of Nrf2 and Sirt3 were significantly elevated in animals subjected to ioversol treatment compared with the control animals (Figures 4(a)–4(c)) (*P* < 0.01). The strongest expression of Nrf2 and Sirt3 was observed in animals subjected to combination treatment with t-BHQ and ioversol. To confirm that t-BHQ treatment activated the Nrf2/Sirt3/SOD2 pathway, the protein levels of Nrf2, Sirt3, Ac-SOD2, and SOD2 were measured. As shown in Figures 5(a) and 5(b), t-BHQ treatment significantly increased the nuclear translocation of Nrf2. Our results showed that while treatment with ioversol upregulated the expression of Sirt3 and SOD2 (Figures 6(a)–6(d)) (*P* < 0.01), treatment with t-BHQ further enhanced the Sirt3 and SOD2 expression in animals subjected to combined t-BHQ+ioversol treatment (*P* < 0.05). We also found that t-BHQ treatment significantly decreased the expression of Ac-SOD2 (*P* < 0.05). Collectively, these results indicated that the nephroprotective role of t-BHQ might be mediated through the Nrf2/Sirt3/SOD2 pathway.

### 3.4. t-BHQ Pretreatment Reduced Oxidative Stress and Increased Cell Viability in HK-2 Cells

**Figure 7:**
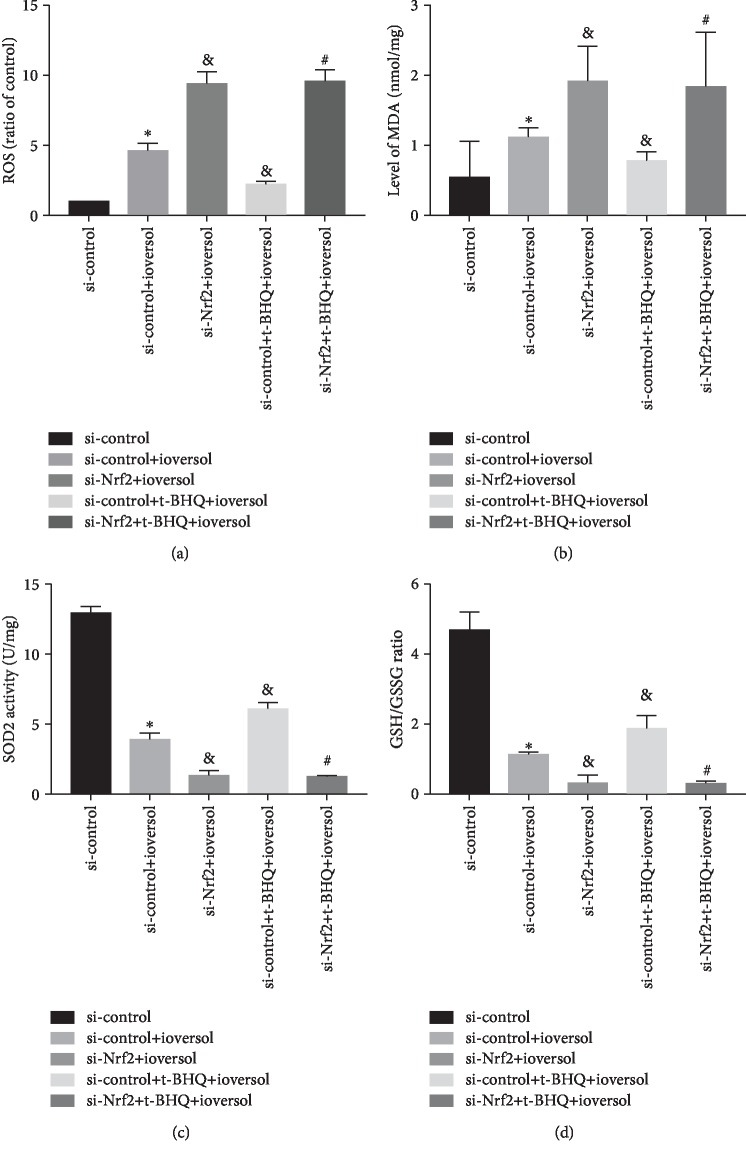
t-BHQ protected against CIN-induced reactive oxygen species in HK-2 cells. (a) ROS level. (b) Malondialdehyde (MDA) level. (c) SOD2 activity. (d) GSH/GSSG ratio. HK-2 cells were infected by si-Nrf2 and then treated with 50 mg/ml ioversol for 24 h to induce oxidative stress. Data were expressed as the mean ± SD. ^∗^*P* < 0.01 versus si-control; ^&^*P* < 0.05 versus the si-control+ioversol group; ^#^*P* < 0.05 versus the si-control+t-BHQ+ioversol group.

**Figure 8:**
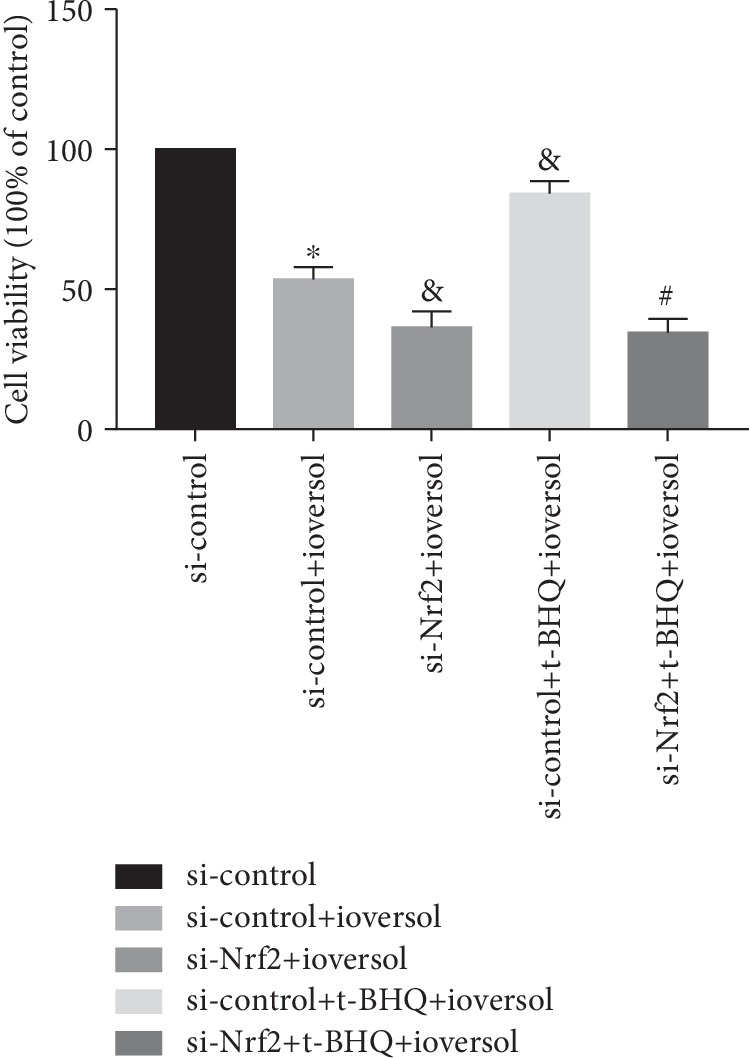
t-BHQ increased cell viability in CIN-induced oxidative injury in HK-2 cells. HK-2 cells were infected by si-Nrf2 and then treated with 50 mg/ml ioversol for 24 h to induce oxidative stress. Data were expressed as the mean ± SD. ^∗^*P* < 0.05 versus the si-control group; ^&^*P* < 0.05 versus the si-control+ioversol group; ^#^*P* < 0.05 versus the si-control+t-BHQ+ioversol group.

We examined the ioversol (50 ml/ml)-induced cellular oxidative stress and its subsequent effect on HK-2 cell viability. Intracellular ROS level, MDA, SOD2 activity, and GSH/GSSG ratio reflect the state of oxidative stress in cells. As shown in Figure 7, ioversol significantly increased the ROS levels and enhanced levels of MDA in HK2 cells. The si-control+ioversol treatment showed a significant lower SOD2 activity and GSH/GSSG ratio than the group treated with si-control alone (*P* < 0.01) (Figures 7(c) and 7(d)). Interestingly, pretreatment with t-BHQ (20 *μ*mol/l) significantly reversed the ioversol-induced oxidative stress injury. As shown in Figure 7(a), cells with silenced Nrf2 showed significant inhibition of the SOD2 activity and GSH/GSSG ratio and increase in the levels of ROS and MDA than cells treated with si-control+ioversol (*P* < 0.05). Furthermore, as shown in Figure 8, the viability of cells treated with the si-control+t-BHQ+ioversol group was significantly higher than the group treated with the si-control+ioversol (*P* < 0.05); in addition, the cell viability of Nrf2-silenced cells was significantly lower than the cells treated with si-control+ioversol (*P* < 0.05). However, pretreatment with t-BHQ neither increased the cell viability of Nrf2 silenced HK2 cells nor did it affect the ROS level, SOD2 activity, MDA content, and GSH/GSSG ratio (Figures 7 and 8).

### 3.5. Nrf2/Sirt3/SOD2 Signaling Pathway Was Involved in the CIN-Induced HK-2 Cell Oxidative Injury

**Figure 9:**
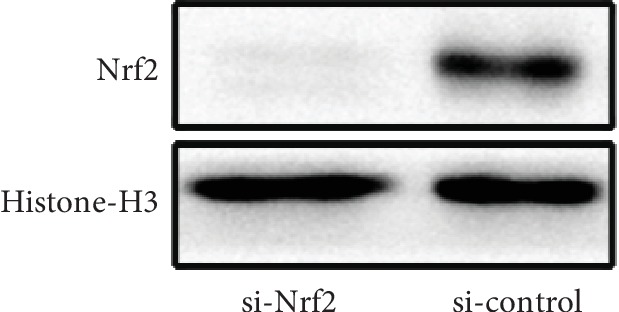
The efficacy of the Nrf2 knockdown by siRNA was measured by western blot.

**Figure 10:**
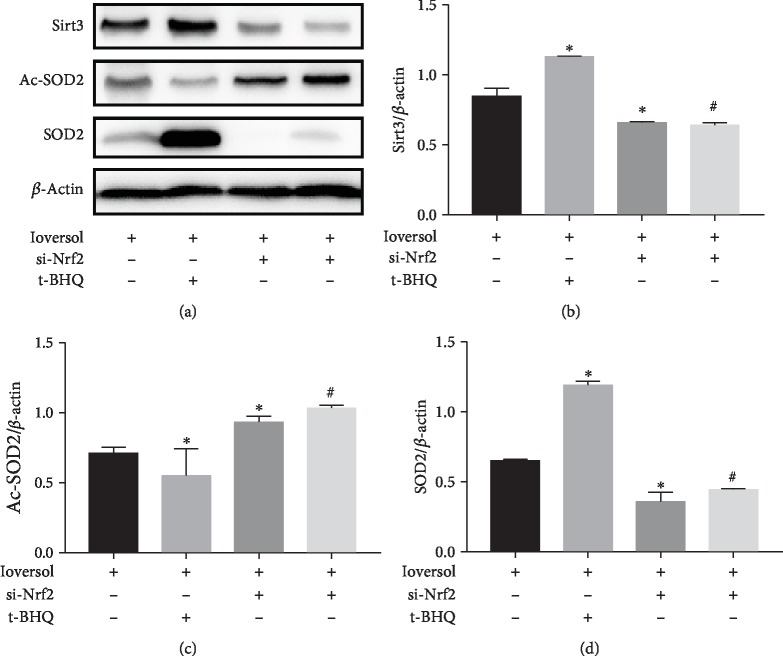
Activation of the Nrf2/Sirt3/SOD2 signaling pathway was essential to the antioxidative effects of t-BHQ in CIN-induced oxidative injury in HK-2 cells. (a) western blot of Sirt3, Ac-SOD2, and SOD2. (b) Quantitative analysis of Sirt3. (c) Quantitative analysis of Ac-SOD2. (d) Quantitative analysis of SOD2. HK-2 cells were infected by si-Nrf2 and then treated with 50 mg/ml ioversol for 24 h to induce oxidative stress. Data were expressed as mean ± SD. ^∗^*P* < 0.05 versus the si-control group; ^&^*P* < 0.05 versus the si-control+ioversol group; ^#^*P* < 0.05 versus the si-control+ioversol+t-BHQ group.

Sirt3 has been reported to play a central role in protection against oxidative stress. To investigate if Nrf2 exerted its protective effects by regulating Sirt3 expression, we transfected Nrf2 siRNA in HK-2 cells and measured the efficiency of the silencing by western blot (Figure 9). Further, as shown in Figures 10(a)–10(d), our results from the western blot analysis showed that ioversol treatment increased the expression of Sirt3 and SOD2 and decreased the expression of Ac-SOD2 in HK-2 cells. Interestingly, pretreatment with t-BHQ enhanced these outcomes (*P* < 0.05). However, ioversol treatment in Nrf2-silenced cells decreased the expression of Sirt3 and SOD2, increased the level of Ac-SOD2, and thereby inhibited the activation of SOD2 (*P* < 0.05). Further, pretreatment with t-BHQ did not modulate the expression of Sirt3, SOD2, and Ac-SOD2 in Nrf2 silenced cells (*P* > 0.05). Collectively, our results suggested that the protective effects of t-BHQ on HK-2 cells against ioversol-induced oxidative stress are possibly mediated by the Nrf2/Sirt3/SOD2 pathway.

## 4. Discussion

CIN is an increasingly common iatrogenic hospital-acquired kidney injury (AKI) occurring after the intravascular administration of radiocontrast agents in diagnostic and interventional procedures [21]. Although it is a reversible and preventable cause of hospital-acquired renal failure, it is correlated with accelerated progression of chronic kidney disease (CKD) [22]. Although the pathophysiological mechanisms of CIN have not been completely understood, hemodynamic perturbations, generation of reactive oxygen species, inflammation, and direct tubular damage have been implicated in its pathogenesis [23]. Studies have shown that oxidative stress plays an important role in the pathogenesis of CIN, and antioxidants may play an important role in the prevention of CIN [24]. Results from the present study showed that contrast agents can result in oxidative injury, exacerbate renal function, and aggravate pathological renal damage. Treatment with ioversol significantly decreased the SOD2 activity and GSH/GSSG ratio and increased the levels of ROS and MDA. The levels of BUN and SCR were higher in the ioversol-treated group than in the control group. Significantly, similar results were obtained *in vitro* using HK-2 cells and were consistent with a previously published study [25]. Like the earlier report, our study also showed that pretreatment with t-BHQ, an agonist of Nrf2, could mitigate oxidative stress and alleviate the progression of AKI. In addition, it also improved cell viability in HK-2 cells. However, there are still some limitations in the present study; we have not performed an experiment to prove t-BHQ could prevent ioversol-induced cytotoxicity in HK-2 cells, but further work will be conducted in the future.

Nrf2 is an important transcription factor belonging to the E26 transformation-specific (ETS) factor family that regulates the cellular resistance to oxidants, including radiocontrast agents [26]. Previous studies have shown that Nrf2 plays a renoprotective role in CIN [25, 27]. Sirt3, which is an NAD^+^-dependent deacetylase localized in the mitochondrial matrix, regulates a variety of cellular processes, including oxidative stress, ATP generation, and energy metabolism. It acts by deacetylating components of electron transport chain (ETC) leading to a lower mitochondrial ROS production and enhancing the activity of ATP synthase [28]. Sirt3 is highly expressed in renal tissues and has been widely studied for its role in acute and chronic kidney diseases [29]. In a murine model of cisplatin-induced acute kidney injury, increased oxidative stress was associated with a reduced level of Sir3 and mitochondrial damage [30]. A recent study showed that ioversol treatment significantly increased the Sirt3 expression in wild-type (WT) mice and HK-2 cells, while Sirt3 deficiency aggravated the contrast-induced acute kidney injury [31]. Although there are scant reports on the role of Sirt3 in contrast-induced AKI, the molecular mechanism of its activation remains unknown. In this study, our results from the western blot and immunohistochemical analysis showed that the contrast-induced acute kidney injury led to the activation of Nrf2 and Sirt3 and that t-BHQ induced the expression of Nrf2 and Sirt3. Importantly, our results were validated by *in vitro* and *in vivo* experiments. Collectively, these results suggest that t-BHQ can alleviate CIN-induced oxidative injury by activating Nrf2-mediated signaling pathway.

Nrf2, known as GA-binding protein, binds with PGC-1*α* to regulate the expression of oxidative genes including Sirt3 [12]. Sirt3 in turn regulates the expression of SOD2 and mediates its protective effects against ROS and mitochondrial oxidative stress [8, 32, 33] by transforming Ac-SOD2 into SOD2 [34]. Further, the Nrf-2a subunit was shown to directly bind to the Sirt3 promoter, and therefore, knockdown or overexpression of Nrf-2 could modulate Sirt3 levels *in vitro* system [12]. Similarly, the AMPK/Nrf2/Sirt3 axis was shown to be involved in the neuroprotective effects of TLB on H_2_O_2_-induced oxidative injury in PC12 cells, wherein the knockdown of Nrf2 led to the inhibition of Sirt3 [35]. Likewise, 17beta-estradiol was shown to enhance nuclear translocation of Nrf2, followed by Sirt3 upregulation and Mn-SOD activation in hUCB-MSCs subjected to high glucose-induced mitochondrial ROS production and autophagy-mediated cell death [36]. Sirt3 eliminates ROS by transforming Ac-SOD2 to SOD2. In this study, we used Ac-SOD2 K68 to assess the activity of Sirt3. t-BHQ treatment increased the expression of Nrf2, Sirt3, and SOD2 and decreased the expression of Ac-SOD2. The activation of Nrf2 increased the expression of Sirt3 and then transformed Ac-SOD2 into SOD2 via deacetylation. Therefore, our data confirms the protective role of Nrf2 and provides novel evidence that Nrf2 plays a crucial role in the activation of the Sirt3/SOD2 signaling pathway in response to contrast-induced oxidative stress and acquired nephropathy.

## 5. Conclusions

Our results showed that pretreatment with t-BHQ can attenuate contrast-induced oxidative stress associated with CIN. The protective effect of t-BHQ could be, at least partly, due to the activation of the Nrf2/Sirt3/SOD2 signaling pathway. Therefore, t-BHQ holds promise as an ancillary therapeutic agent for the clinical management of CIN.

## Data Availability

The data used to support the findings of this study are available from the corresponding authors upon request.
